# Editorial: 15 years of Frontiers in Cellular Neuroscience: the dual role of microglia in (neuro)inflammation

**DOI:** 10.3389/fncel.2025.1567407

**Published:** 2025-02-11

**Authors:** Antonio J. Herrera

**Affiliations:** ^1^Institute of Biomedicine of Seville (IBIS)-Hospital Universitario Virgen del Rocío/CSIC/University of Seville, Seville, Spain; ^2^Department of Biochemistry and Molecular Biology, Faculty of Pharmacy, University of Seville, Seville, Spain

**Keywords:** dual role, microglia, neurodegeneration, neuroinflammation, neuroprotection

**Figure 1:**
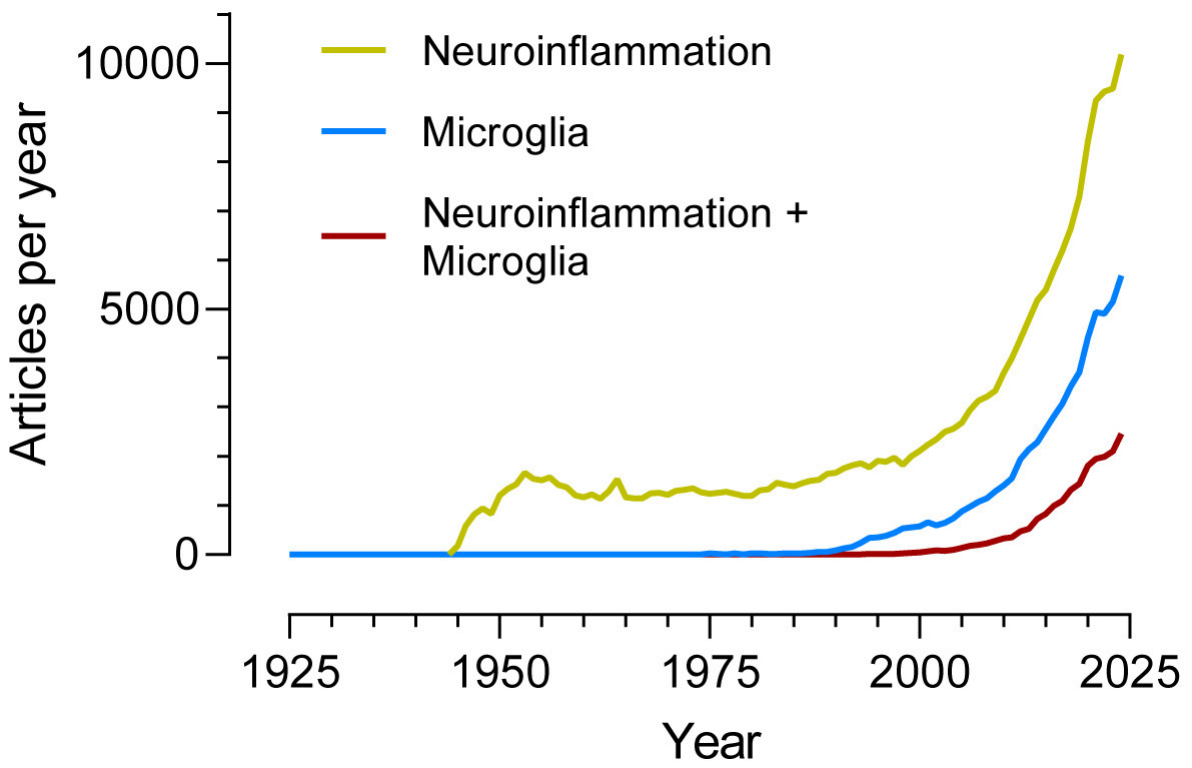
Number of articles per year (1925–2024) indexed in the PubMed database and identified using the search terms “Neuroinflammation”, “Microglia”, and “Neuroinflammation + Microglia”.

If the search terms “Neuroinflammation” and “Microglia” are entered independently into PUBMED, and the number of published articles per year that include them is plotted (see Figure 1), a notable increase is observed that coincides approximately with the turn of the century (and millennium). The search “Neuroinflammation + Microglia” also shows a clear increase delayed, however, by about 10–15 years compared to the previous two.

Interest in microglial cells as agents involved in neuroinflammation is recent but growing rapidly, although we still do not know if their overall action is protective or damaging to neurons; the search “Neuroinflammation + Microglia + Protective” yields approximately the same result as “Neuroinflammation + Microglia + Damaging.”

Microglia act as homeostatic cells in the central nervous system. In recent years, research on microglia has focused on defining their role in brain physiology, both in health and disease. What has been found exceeds previous expectations, as microglial cells actively participate not only in surveillance tasks of nervous tissue but also in different aspects of neurogenesis, neuronal connectivity, synaptic control (through pruning, for example), and myelination, among others.

When nervous tissue is threatened or damaged, microglia initiate a beneficial acute inflammatory process characterized by the release of inflammatory mediators and phagocytosis of cellular debris. This process ends after eliminating the threat (pathogens, for example) and repairing the damage. However, the alteration of this physiological self-limited acute healing inflammatory process, for reasons not yet well-understood, leads to a self-sustained neuroinflammatory state. In this state, inflammation produces damage, and damage, in turn, produces more inflammation in a continuous cycle.

The Research Topic “*15 years of Frontiers in Cellular Neuroscience: the dual role of microglia in (neuro)inflammation*” explores some of the microglia-mediated processes that influence brain and neuronal homeostasis, as well as the factors that modify them, such as stress.

Traumatic brain injury (TBI) affects tens of millions of people worldwide, creating an unbearable burden for public health systems and for affected individuals and families. This is due to the high number of hospitalizations and deaths it causes, as well as the long-lasting functional deficits (cognitive, psychiatric, and physical) experienced. Boland and Kokiko-Cochran review the role of microglia in TBI, their relationship with other cell types (neurons, astrocytes, and peripheral immune cells), and methods to manipulate them, focusing on the use of colony-stimulating factor 1 receptor (CSF1R) inhibitors and the results obtained in studies using these inhibitors in TBI.

As TBI alters baseline levels of stress hormones, as well as the response to external stressors (including medical-related psychological stress), different natural and synthetic glucocorticoids (GC) have been used to treat some of the chronic side effects associated with persistent neuroinflammation. Taylor and Kokiko-Cochran review the stress response and glucocorticoid signaling in the context of TBI. They suggest that the therapeutic approach based on GC and manipulation of their receptors (GR) should be modified to be specific for each cell type, focusing on their effects on microglia and the associated neuroinflammation.

Neurodegenerative diseases affect a growing number of individuals worldwide. Alzheimer's disease alone, the most prevalent among them, is estimated to affect more than 100 million people by 2050. While TBI is an enormous public health problem, it is difficult to express in words the devastating impact that neurodegenerative diseases have today and will have in our near future. It is a fact that microglial functions are dysregulated in these diseases, which explain why many of the scientific articles counted in the figure try to understand how this alteration occurs and find ways to prevent or even reverse it.

Pro-inflammatory microglia require high amounts of glucose to function. Chung et al. study the role of a homeostatic protein, the Regulator of G-protein Signaling 10 (RGS10), in maintaining microglial homeostasis under conditions of hyperglycemia and neuroinflammation related to the disease. Their results support that RGS10, abundant in microglia and downregulated in neurodegenerative diseases, is a negative regulator of microglial activation, which develops with the production of pro-inflammatory cytokines and reactive oxygen species.

To date, treatments available for Alzheimer's disease are purely palliative (fundamentally inhibiting acetylcholinesterase and trying to keep excitotoxicity under control), although recent advances have been made using immunotherapy. Karimi Roshan et al. propose a new therapeutic approach by adapting methods used in the treatment of various forms of cancer to Alzheimer's disease: Radiation therapy, specifically through the adaptation of boron neutron capture therapy (BNCT). Their results show a strong pro-inflammatory induction of microglia, with induced phagocytosis that helps eliminate beta-amyloid. Of great interest is that this therapy seems to induce microglia transiently, without long-term alterations, avoiding the self-sustained neuroinflammatory state in which inflammation produces damage and damage produces inflammation endlessly.

